# Methods and Applications in Trapped Charge Dating

**DOI:** 10.3390/mps3010024

**Published:** 2020-03-24

**Authors:** James K. Feathers

**Affiliations:** Department of Anthropology, University of Washington, Seattle, WA 98195, USA; jimf@uw.edu

**Keywords:** luminescence, electron spin resonance, chronology, earth sciences, archaeology

## Abstract

Trapped charge dating is a commonly used chronological tool in Earth Sciences and Archaeology. The two principle methods are luminescence dating and electron spin resonance. Both are based on stored energy produced by the absorption of natural radioactivity in common minerals such as quartz and feldspars and in some biological materials such as tooth enamel. Methodological developments in the last 20 years have substantially increased accuracy and precision. This essay introduces a compilation of papers that offers a taste of recent research into both method and application.

Trapped charge dating is a fast developing field that provides chronological and other information, principally in the geological and archaeological sciences. There are two main methods, luminescence dating and electron spin resonance (ESR) dating. Both are based on the storage of energy in certain materials as a function of natural radioactivity. When radiation impinges on such material, for example, quartz and feldspar minerals or tooth enamel, ionization produces detached electrons and electron vacancies, that can move about the crystal lattice. Most of them recombine and return to the ground state, but some become attracted to localized charge deficiencies associated with defects in the crystal. They are “trapped” at these defects until heat or sunlight provides sufficient energy to release them. The trapped charge builds up as a function of time, according to the rate of absorbed radiation. The amount of stored energy is thus proportional to the time when the material was last exposed to heat or sunlight (which cleans out the traps), or in the case of organic material such as teeth and shell, the time of crystalline formation. The accumulated energy in the traps can be related to radiation by calibration with artificial radiation in the laboratory, resulting in a quantity called equivalent dose, which is the amount of radiation dose necessary to produce the amount of trapped charge. Dividing the equivalent dose by the natural dose rate yields an age, or the time since the traps were last emptied. Thus, the time can be determined when ceramics or rocks were last heated, when sediments were last exposed to light (at time of burial) or when teeth or shells formed. This is possible because the long half-lives of the principle components of natural radioactivity mean that the dose rate is, in most cases, effectively constant through time. 

Luminescence methods, which are mainly applied to quartz or feldspars, measure the trapped charge by stimulating with heat or light to release the charge. Recombination then produces light, called luminescence, whose intensity is proportional to the amount of stored energy. When stimulated by heat, the resulting signal is called thermoluminescence (TL). When stimulated by light (or more properly by photons), the resulting signal is called either optically stimulated luminescence (OSL)when the stimulation is with visible light or infrared stimulated luminescence (IRSL) when stimulation is with infrared light. ESR measures the trapped charge directly in the traps by inducing absorption resonance between two spin states by microwave radiation in a magnetic field. The amplitude of the resonance is proportion to the number of trapped electrons. ESR if often applied to tooth enamel and shells but also to quartz. Reviews of different aspects of both methods can be found in Rink and Thompson [1].

While these methods first developed during the last half of the 20th century, significant developments in instrumentation, method and application have occurred during the last 20 years, making luminescence, at least, the second most utilized chronological metric in Quaternary science, after radiocarbon dating. Applications have spread beyond dating to studies of provenience, exhumation rates and erosion rates, but have included novel extensions in dating, such as surface dating of rocks, complementing cosmogenic dating methods. Method improvement for both equivalent dose and dose rate have improved both accuracy and precision. 

This compilation is an eclectic assortment of papers, which, while not fully representative of the wide scope of trapped charge methods, gives a taste of the range of methods and applications.

Two of the papers deal with techniques, the improvement of which has become necessary as the range of applications broaden. In archaeological and cultural heritage studies, minimum destruction of the record is imperative. Sample collection needs to be done in the least invasive way. Nelson et al. [2] explored the range of options in collecting sediment samples for luminescence measurements by coring, obviating the need for expensive and destructive excavation. Coring is also important for reaching otherwise hard-to-get targets, such as marine and ocean sediments. Obtaining equivalent dose values on single grains has become a major tool in luminescence dating for evaluating the integrity of deposits and dealing with mixed age sediments. This has also put a premium on getting additional information, such as composition (for dose rate determinations, among other things) and shape, from the individual grains measured. Doverbratt and Alexanderson [3] detail methods for transferring grains from the single-grain disks used to measure luminescence to other media, so that information from the same grains can be obtained.

While most research is directed toward determining an accurate measure of the equivalent dose, dose rate measurements are equally important, even if given less attention. Hood and Highcock [4] consider problems in determining the dose rate in complex environments, which are often encountered at archaeological sites. They demonstrate the use of DosiVox, a new computer program for reconstructing the radioactive environment, in this case for pottery vessels from Egyptian monuments. Blackwell et al. [5] discuss the need for intensive sampling to disentangle varied and high dose rates to tooth fragments, in the context of ESR dating, in cave sediments in the Caucasus of southern Russia.

A constant theme in trapped charge studies is the attempt to extend the possible dating range, both for very young samples and for very old samples. Spencer et al. [6], in a study of active fluvial processes in the Amazon River catchment, demonstrated the possibility of obtaining OSL ages as young as 13-14 years. This allows one to understand fluvial dynamics in the context of recent land use changes. The upper dating range of trapped charge method is ultimately defined by saturation of the traps. This occurs later for ESR than luminescence, and later for IRSL of feldspars than the OSL of quartz. The problem of saturation in quartz and the resulting underestimation of age is explored by Groza-Săcuciu et al. [7] in their study of Romanian loess, in the context of using different grain sizes of quartz. This problem is far from resolved in luminescence studies, and the authors present a systematic inquiry into various possible causes of the age underestimation of fine-grain compared to coarse-grain quartz for equivalent dose values of more than 50 Gy.

IRSL of feldspar saturates at a higher level than quartz and so is increasingly turned to for dating older sediments. Feldspar, however, suffers from an athermal loss of signal over time, called anomalous fading. While corrections for fading are possible, they work less well for older sediments. A non-fading signal has been documented for a protocol called post-IRSL IRSL (pIRSL), where a higher temperature stimulation follows a lower temperature one. The higher temperature stimulation taps traps less likely to fade. Zhang and Li [8] present a comprehensive review of the various pIRSL methods and also introduce the possibility of standard growth curves (luminescence versus dose) for feldspars.

Standard growth curves (SGC) are also discussed for quartz by Hu et al. [9]. They construct different SGCs for different groups of quartz grains, measured at the single-grain level. Dating older samples with quartz depends on isolating those quartz grains with high characteristic doses (which define the shape of saturating exponential functions).

The intensity of luminescence and ESR signals has also proved a fruitful subject of study. The intensity of quartz is known to increase with the number of cycles of exposure and burial experienced by the sediment. Thus, samples close to the bedrock source are less sensitive than those which have been transported a long way from the source. Sawakuchi et al. [10] have used differences in sensitivity to differentiate provenience of Amazon sediments. In this paper, they test methods for streamlining the measurement procedure, allowing expanded measurement probabilities, including in situ measurements with portable equipment. Tsukamoto et al. [11] show that the intensity of the ERS signal from oxidized iron increases with age and use this information to date gut strings from historic plucked instruments. For instruments of a known age, the method can determine if the strings are as old as the instrument.

Finally, Zhang et al. [12] discuss the problems of dating fluvial terraces in China. They show that one sample from each of the terraces is not sufficient. Rather, systematic sampling of each terrace is required to see how the terraces evolve, how they relate to each other, and to determine sedimentation rates.
